# *Haloferax volcanii*, as a Novel Tool for Producing Mammalian Olfactory Receptors Embedded in Archaeal Lipid Bilayer

**DOI:** 10.3390/life5010770

**Published:** 2015-03-09

**Authors:** Simona Lobasso, Rita Vitale, Patrizia Lopalco, Angela Corcelli

**Affiliations:** 1 Department of Basic Medical Sciences, Neurosciences and Sensory Organs, University of Bari “Aldo Moro”, Piazza Giulio Cesare 11, I-70124 Bari, Italy; E-Mails: simona.lobasso@uniba.it (S.L.); rita.vitale@uniba.it (R.V.); patrizia.lopalco@uniba.it (P.L.); 2 Institute for Chemical-Physical Processes, National Research Council (IPCF-CNR), Bari Unit, via Orabona 4, I-70126 Bari, Italy

**Keywords:** extremely halophilic microorganisms, archaeal lipids, archaeonanosomes, olfactory receptors, biosensors

## Abstract

The aim of this study was to explore the possibility of using an archaeal microorganism as a host system for expressing mammalian olfactory receptors (ORs). We have selected the archaeon *Haloferax volcanii* as a cell host system and one of the most extensively investigated OR, namely I7-OR, whose preferred ligands are short-chain aldehydes, such as octanal, heptanal, nonanal. A novel plasmid has been constructed to express the rat I7-OR, fused with a hexahistidine-tag for protein immunodetection. The presence of the recombinant receptor at a membrane level was demonstrated by immunoblot of the membranes isolated from the transgenic archaeal strain. In addition, the lipid composition of archaeonanosomes containing ORs has been characterized in detail by High-Performance Thin-Layer Chromatography (HPTLC) in combination with Matrix-Assisted Laser Desorption Ionization—Time-Of-Flight/Mass Spectrometry (MALDI-TOF/MS) analysis.

## 1. Introduction

Olfactory receptors (ORs) are members of the large super family of G-protein coupled receptors (GPCRs) [1,2,3]. GPCRs are integral membrane proteins that possess seven membrane-spanning domains or transmembrane helices [4]. ORs’ main biological function is the molecular recognition of odorant molecules that can easily disperse into the air. Detection of odors results from the association of small hydrophobic molecules with the specific ORs, located in the cilia of olfactory sensory neurons in the nasal olfactory epithelium [5]. Although neural pathways leading to odor recognition in the brain are well known, information on the detailed molecular structures of ORs is quite limited.

In the present study, we have used the rat I7-OR as a model to investigate ORs expression in archaeal microorganisms, as it is one of the most extensively investigated OR. It has been cloned, expressed in neurons, functionally characterized as activated by short-chain aldehydes, such as octanal, heptanal, and nonanal [6,7], and studied by computational modeling [8,9].

Despite many research groups having made several attempts to express ORs in heterologous cell systems (for example, in yeast [10], in insect Sf9 cell line [11], in HEK293 cell line [12,13], in Hela/olf cell line [14]), improving those systems is still a matter of research. Here, we have explored the possibility of using the archaeon *Hfx. volcanii* as a host cell system for heterologous expression of ORs.

As some archaeal microorganisms can express large amounts of seven transmembrane helix proteins, such as bacteriorhodopsin, they can be considered good candidates to host ORs. On the other hand, in the past, investigations on the possibility of using archaea as a production system for human β_2_-adrenergic receptor and other eukaryal GPCRs have been carried out [15,16].

Our present biotechnological approach offers the possibility to exploit *Haloferax* as a host for heterologous expression of eukaryotic membrane proteins with the perspective to isolate membranes embedding ORs, namely archaeonanosomes, to be used as sensing element of a bioelectronic nose.

*Hfx. volcanii* is an extremely halophilic archaeal microorganism belonging to the family *Halobacteriaceae*, easy to culture in the laboratory, whose genome has been sequenced [17]. It grows, like other haloarchaea, in high salt concentrations (2 to 4 M NaCl), and to cope with the osmotic potential of such environments, it accumulates high intracellular concentration of K^+^ ions [18]. *Hfx. volcanii* is widely regarded as the best-equipped organism for archaeal genetics, because of several selectable markers and plasmids for transformation and gene knockout [19,20], as well as reporter genes [21] and inducible promoter [22].

Like other salt-loving archaeal microorganisms typically found in coastal saltern ponds and hypersaline lakes, the plasma membranes of *Hfx. volcanii* are characterized by the presence of beautiful red-orange pigments and anionic phospholipids and glycolipids. The pigments help to screen out UV radiation and protect the cells from the harmful effects of sunlight, while the negatively charged groups of phospholipids and glycolipids confer a high negative charge density to the membranes.

The unique structural features of the archaeal polar membrane lipids, that is, the *sn*-glycerol-1-phosphate backbone, isoprenoid hydrocarbon chains and ether linkages, are in striking contrast to the bacterial and eukaryotic characteristics of the *sn*-glycerol-3-phosphate backbone, fatty acid chains, and ester linkages. The diether lipid core that forms the basis for most polar lipids present in the family *Halobacteriaceae* is 2,3-di*-O-*phytanyl-*sn*-glycerol (C_20_, C_20_), also called archaeol [23]; in some haloalkaliphile and *Halococcus* species, C_20_, C_25_-, and C_25_, C_25_-diether variants of the diphytanylglycerol diether lipid core were also identified [24].

It is well known that membranes of Archaea are very stable over various salt concentrations and pH values. In fact, ether linkages of archaeal lipids are more stable than esters over a wide range of pH, and the branching methyl groups of the archaeol help to reduce both crystallization (membrane lipids in the liquid crystalline state at ambient temperature) and membrane permeability (steric hindrance of the methyl side groups). The stereochemistry of glycerol backbone would impart resistance to attack by phospholipases produced by other organisms. The saturated alkyl chains would impart stability towards oxidative degradation, particularly in halophiles exposed to sunlight and air.

Thus, due to their chemical-physical properties, archaeal lipids are considered an ideal biomaterial to prepare lipid matrix for biosensor devices incorporating membrane proteins, such as ORs.

Here we have isolated, for the first time, archaeal membrane domains containing ORs, which may represent a rich source of inspiration for the conceptual development of innovative assemblies and biomaterials, such as a nanovesicle-based bioelectronic nose that could mimic the receptor-mediated specificity of olfactory system.

In the past, patches of archaeal plasma membranes, isolated from the extremely halophilic microorganism *Halobacterium salinarum* (*i.e.*, purple membrane, containing bacteriorhodopsin, a seven transmembrane protein-like ORs), have been demonstrated to wrap and functionally interact with single walled carbon nanotubes (SWNTs) [25]. Then, SWNTs have been also surface functionalized with an archaeal glycolipid similar to the main glycolipid of *Hfx. volcanii* [26]*.* It has been demonstrated that the archaeal glycolipid molecules wrap the SWNTs, self-assembling onto their walls by means of glucose headgroups [26]*.* Such a specific supramolecular binding and the consequent direct electron interfacing between the two moieties suggest that such hybrid materials can represent a promising key element in the fabrication of bionanoelectronic devices.

## 2. Experimental Section

### 2.1. Materials

Unless stated otherwise, kits and reagents for molecular biology techniques were from Invitrogen (Carlsbad, CA, USA). Restriction enzymes used for molecular cloning were from New England Biolabs (Ipswich, MA, USA). DNA size markers and protein size marker (Prestained SDS/PAGE Standard, Low Range) were from Bio-Rad (Hercules, CA, USA). High-Performance Thin-Layer Chromatography (HPTLC) plates (Silica gel 60A, 10 cm × 20 cm, layer thickness 0.2 mm, purchased from Merck) were washed twice with chloroform/methanol (1:1, *v*/*v*), and then activated at 120 °C before use. The matrix 9-aminoacridine hemihydrate for Matrix-Assisted Laser Desorption Ionization—Time-Of-Flight/Mass Spectrometry (MALDI-TOF/MS) analysis was obtained from Acros Organics. Unless stated otherwise, chemicals were from Sigma-Aldrich (St. Louis, MO, USA). All organic solvents used were commercially distilled and of the highest available purity.

### 2.2. Strains and Culture Condition.

*Hfx. volcanii* strain WR-340 was kindly provided by Jorg Soppa. Cells were grown in the light in an orbital shaker at 160 rpm and 37 °C, in a complex liquid medium whose composition has been previously described [27]. Plates were prepared adding 15 grams of agar per liter to the liquid medium. *E. coli* strain JM109 (Promega, Fitchburg, WI, USA) was grown in SOB^+^ complex medium [28] supplemented with 0.1 g/L ampicillin, when necessary.

### 2.3. Extraction of Total Norway Rat Genomic DNA

Total genomic DNA was extracted from 200 mg of rat-frozen heart (*Rattus norvegicus*) using the Trizol Reagent (Invitrogen, Carlsbad, CA, USA) and resuspended in TE buffer (10 mM Tris/Cl and 1 mM EDTA, pH 7.5). I7-OR receptor gene was amplified by PCR on total genomic DNA using I7 for and I7His rev primers (Table 1). The reverse primer I7His rev was designed to introduce the codons for a six Histidine epitope tag to the *C*-terminus of the protein. The PCR product was then purified, sequenced, and digested with *NheI* and *KpnI* restriction enzymes at 37 °C for two hours.

**Table 1:** Sequences of oligonucleotides used in this study.

Name	Sequence (5'→3')
I7 for	gctagcatggagcgaaggaaccacag
I7 His rev	ggtaccctaaccaattttgctgcctttgt
Linker 1	catgggggctagcgattgcgatccgattcggtac
Linker 2	cgaatcggatcgcaatcgctagccc

### 2.4. Vectors

pNP-I7 vector, used to express the rat olfactory receptor I7-OR (entry M64386 in GenBank) in *Hfx. volcanii*, was based on the shuttle vector pNP-8, kindly provided by Jorg Soppa. The pNP-8 vector, a derivative of pSD1-R1/6 vector [16], was modified for the addition of the new restriction site of *Nhe I* enzyme. Briefly, 50 pmol of each of the two oligonucleotides “Linker 1” and “Linker 2” (Table 1) were mixed and incubated at 96 °C for 2 h. The mixture was diluted to 100–500 femtomoles/µL and co-incubated with 500 ng pNP-8 vector; then the plasmid was extracted after the propagation in *E. coli*, digested with *NcoI* and *KpnI* restriction enzymes for 2 h at 37 °C, and directly purified from agarose gel. After 24 h of incubation at room temperature, 5 µL of the ligation mixture was added to 100 µL of *E. coli* JM109 competent cells. The vector, extracted from *E. coli* competent cells, was then digested by *NheI* and *KpnI* restriction enzymes at 37 °C for2 h, ligated with the I7-OR receptor gene purified PCR product, and then digested with the *NheI* and *KpnI* restriction enzymes. The ligation mix was used to transform *E. coli* competent cells. Further experiments of PCR colony and sequencing were used to confirm both the sequences of I7-OR and pNP-I7 vector. Plasmid DNA was then extracted from *E. coli* cultures and used to transform *Hfx. volcanii* cultures.

### 2.5. Transformation of Hfx. volcanii

Transformation of *Hfx. volcanii* was performed as previously described [27]. Briefly, 1 mL of a stationary phase starter culture was inoculated in 50 mL of complex liquid medium, and incubated in a 250 mL flask at 37 °C in an orbital shaker (150 rpm). Transformed cells were plated and plates were incubated at 42 °C for 15–20 days. Transformed colonies were selected on the basis of the resistance to the antibiotic novobiocin (1 µg/mL).

### 2.6. Reverse Transcription—Polymerase Chain Reaction (RT-PCR)

Total RNA was extracted from frozen tissue by the Trizol one-step procedure (Invitrogen, Carlsbad, CA, USA) in accordance with the manufacturer’s instructions. To assess the quality of the extraction, RNA was separated on formaldehyde-containing agarose gels. RT-PCR with I7-OR specific primers I7 for and I7 His-rev (Table 1) was undertaken to assess the expression of I7-OR mRNA in *Hfx. volcanii*. Negative controls for the presence of remaining DNA were provided by RT-PCR with the same primers performed on non-reverse transcribed mRNA.

### 2.7. Sequencing

All DNA sequences were determined by an *ABI PRISM*^®^ *310* Genetic Analyzer (Applied Biosystems, Waltham, MA, USA).

### 2.8. Isolation of Hfx. volcanii Membranes

Cells from liquid cultures of transformed *Hfx. volcanii* strain were collected by centrifugation (8000× *g* for 15 min at 4 °C), washed, and resuspended in a buffer containing 3.4 M NaCl, 0.050 M Tris/Cl, pH 7.4. A dialysis bag (cut-off 12–14 kDa) was filled with a concentrated suspension of cells in the presence of DNase I and left under stirring at 4 °C overnight against 40 volumes of Tris/Cl 0.050 M, pH 7.4. The lysate was centrifuged (30,000× *g* for 40 min) and the resulting pellet, containing the membranes, was washed twice with buffer A (0.1 M NaCl, 0.050 M Tris/Cl, pH 7.4). Isolated membranes were resuspended in the same buffer and stored at −20 °C until further use. The protein concentration of the membrane preparation was determined using the Bradford Protein Assay (Biorad, Hercules, CA, USA).

### 2.9. Immunoblot Analysis

Proteins of the membrane fraction were separated by electrophoresis on 12% sodium dodecyl sulphate (SDS) polyacrylamide gels, and electrotransferred onto Hybond ECL nitrocellulose membrane (Amersham Pharmacia Biotech Europe, Amersham, UK). Positive and negative control lysates of *E. coli* were purchased from Pierce (Rockford, IL, USA). The membrane was blocked for 1 h at room temperature with the Blocking Reagent (3% (*w*/*v*) bovine serum albumin (BSA) in TBS buffer) supplied with the mouse monoclonal IgG1 Anti His Antibodies (Penta His HRP Conjugate Kit, 5PRIME GmbH, Hamburg, Germany). After removing the blocking reagent, membranes were incubated overnight at 4 °C with the mouse monoclonal IgG1 Anti-His Antibody (1:500 concentration) (5 PRIME GmbH, Hamburg, Germany). After washing, blots were revealed using the enhanced chemiluminescence (ECL) detection kit from Amersham Pharmacia Biotech Europe.

### 2.10. Lipid Extraction and HPTLC Analysis

Total lipids were extracted from engineered membranes isolated from *Hfx. volcanii* using the Bligh and Dyer method, as modified for extreme halophiles [29]; the organic extracts were carefully dried under N_2_ before weighing and then dissolved in chloroform. Total lipid extracts were analyzed by HPTLC plates with Solvent A (chloroform/methanol/90% acetic acid, 65:4:35, *v*/*v*/*v*). Lipid detection was carried out by spraying with 5% sulfuric acid in water, followed by charring at 180 °C for 7–8 min [29]. The lipid species were identified by comparing the retention factor values obtained with those of archaeal lipid standards [30,31].

### 2.11. MALDI-TOF/MS Lipid Analysis

Lipids of intact engineered membranes have been directly analyzed by MALDI-TOF/MS analysis, following a procedure recently described. The lipid extraction steps have been avoided and membranes have been directly loaded over the MALDI target [32]. MALDI-TOF mass spectra of membranes were acquired on a Bruker Microflex mass spectrometer (Bruker Daltonics, Bremen, Germany). The system utilizes a pulsed nitrogen laser, emitting at 337 nm, the extraction voltage was 20 kV. For each mass spectrum, 999 single laser shots were averaged. The laser fluence was kept at about 60% of maximum value to have a good signal-to-noise ratio. Spectra were acquired in negative ion mode. Spectral mass resolutions and signal-to-noise ratios were determined by the software for the instrument, “Flex Analysis 3.3” (Bruker Daltonics).

## 3. Results and Discussion

In order to express ORs in the membranes of haloarchaea, we adapted strategies that had been previously developed for heterologous expression of other GPCRs in *Halobacterium salinarum* and *Haloferax volcanii* [15,16]. In particular in the present study a novel plasmid shuttle vector, based on a plasmid previously utilized to produce rat neuromedin K3 receptor [16], has been constructed and used to express the I7-OR in *Hfx. volcanii.*

The plasmid utilized the strong aloarchaeal synthetic promoter PrR16 and contained a haloarchaeal replication origin; the selection of the plasmid was based on the presence of the mutated *GyrB* sequence, which allowed growth of transformed microorganisms on media containing the antibiotic novobiocin [16].

In the novel plasmid, named pNP-I7, both the coding regions for neuromedin K3 receptor and the enzyme dihydrofolate reductase were replaced by rat I7-OR sequence, as described in detail in the Methods Section.

**Figure 1:**
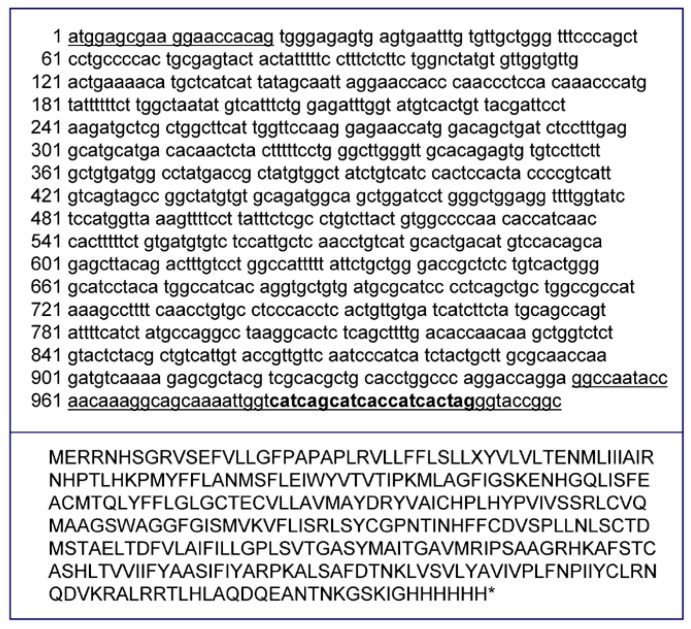
Nucleic acid and amino acid sequences of the olfactory receptor I7-OR from *Rattus norvegicus.* The 6×His tag (*in bold*) was added at the C-terminus of I7-OR sequence for immunological detection. The I7-OR fusion gene (about 1 Kb) was generated by PCR using genomic DNA, extracted from rat heart*,* and the underlined oligonucleotides.

Furthermore, in order to express a receptor having a *C*-terminal hexahistidine (6×His) tag useful for protein immunodetection, a sequence encoding a 6×His peptide was added downstream of the I7-OR coding region in the novel pNP-I7 vector. Figure 1 shows the nucleic acid and amino acid sequences of the I7-OR from *Rattus norvegicus.*

*Hfx. volcanii* cells were transformed with the vector pNP-I7 containing the coding gene for I7-OR. Stable propagation of the plasmids including the I7-OR gene was verified by plasmid re-isolation from transgenic cultures after more than 10 generations and characterization of gene integrity by PCR analysis.

Transformation of the *Hfx. volcanii* strain with this expression vector gave rise to a very small number of selectable novobiocin-resistant clones, which were identified by colony screening (not shown).

**Figure 2:**
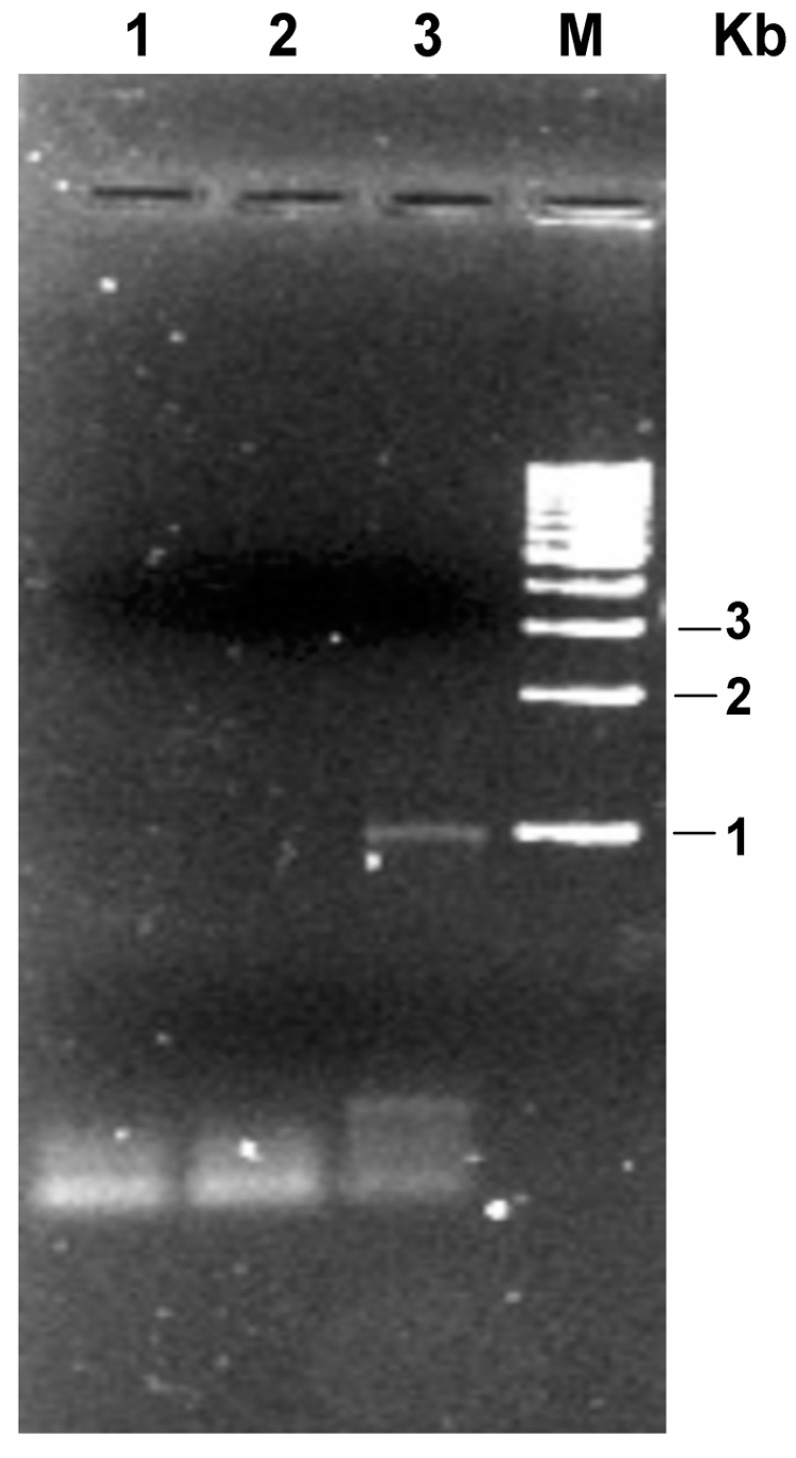
mRNA of the rat I7-OR in transformed *Hfx. volcanii* strain. Total RNA was extracted from *Hfx. volcanii* cells transformed with pNP-I7 expression vector and subjected to RT-PCR using specific primers. (1) PCR blank; (2) PCR negative control (absence of RT enzyme in the mixture); (3) cDNA of *Hfx. volcanii*; (M) molecular mass marker.

Figure 2 shows the RT-PCR analysis conducted on RNA extracted from the transformed strain: it can be seen that RT-PCR product for the I7-OR was found at expected size (about 1000 bp). No such transcript was detected in a negative control.

**Figure 3:**
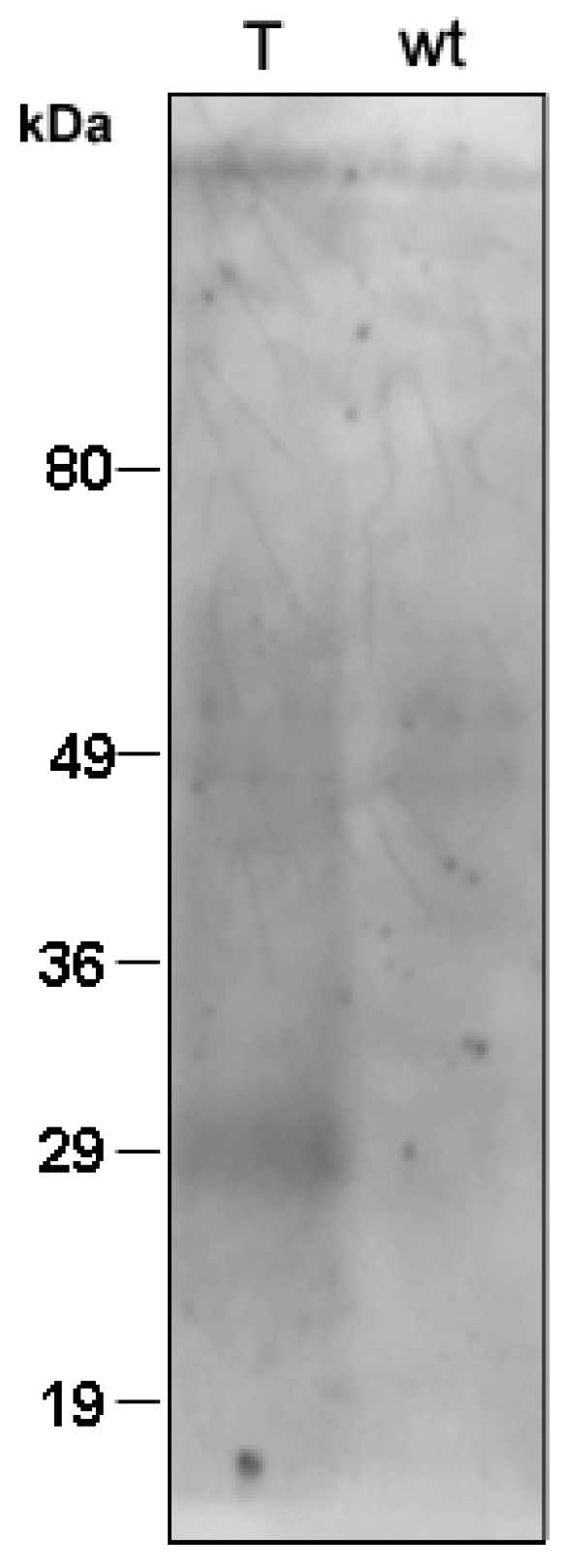
Localization of reporter construct in transgenic cell cultures. Total proteins from transformed (*T*) and wild-type (*wt*) cells were precipitated with TCA and analysed by 12.5% SDS-PAGE, followed by Western blotting using anti-6×His antibodies. Fragment sizes of the molecular mass marker are indicated on the left.

To examine the presence of the I7-OR protein in *Hfx. volcanii* transformed cells, firstly Western blot of total cell proteins, prepared from both wild-type and transformed clones, was developed with an anti-6×His antibody (Figure 3). No immunoreactivity was detected in control sample from wild-type cells, while one immunoreactive band was observed in the case of transformed cells. It can be observed that the apparent molecular weight of the recombinant protein expressed by transformed *Hfx. volcanii* (about 30 kDa) is lower than the calculated molecular weight for the I7-OR protein having 6×His tag in its amino acid sequence (39 kDa).

In order to examine the subcellular localization of the produced protein in the microorganism, plasma membranes were isolated from both wild-type and transformed cultures of *Hfx. volcanii* after cell disruption by hypoosmotic shock.

**Figure 4:**
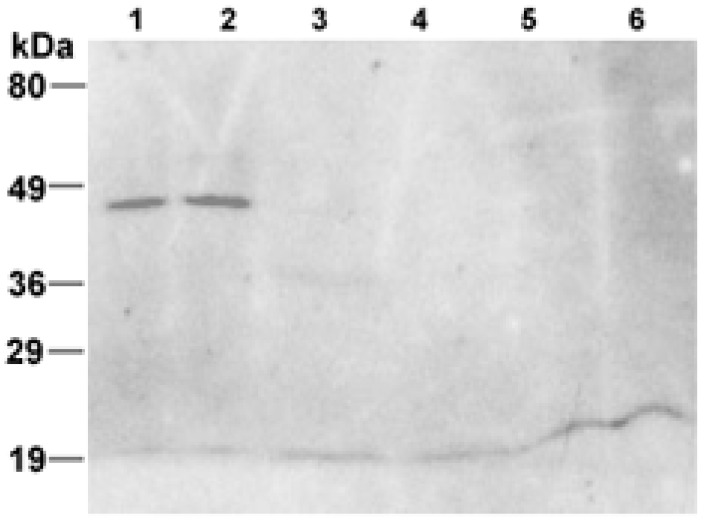
Immunoblot analysis of the I7-OR in membranes isolated from transformed and wild-type cells. (1) and (2) membranes isolated from transformed cells (80 and 100 µg, respectively); (3) positive control, lysate of *E. coli* culture expressing 6×His-tagged urate oxidase (35 kDa) protein; (4) negative control, lysate of *E. coli* culture not expressing any 6×His-tagged protein; (5) and (6) membranes isolated from wild-type cells (80 and 100 µg, respectively). Fragment sizes of the molecular mass marker are indicated on the left.

Figure 4 shows the Western blot of both the membrane preparations performed with the anti-6×His antiserum. One immunoreactive band was visible at approximately 45 kDa exclusively in the membrane fraction isolated from transformed cultures (Figure 4, Lanes 1 and 2). No immunoreactivity was detected in the membranes isolated from wild-type strain (Figure 4, Lanes 5 and 6). Furthermore, one minor immunoreactive band was visible in the positive control at the expected size (Figure 4, Lane 3), whereas no bands were detected in the negative one (Figure 4, Lane 4). However, the size of the immunoreactive band observed in the membrane fraction isolated from transformed culture does not perfectly match with the expected molecular weight of the engineered protein.

The discrepancies in size of the detected I7-OR protein in whole cell extracts *versus* isolated membranes (Figure 3 and Figure 4) could be explained considering that, in the presence of a different detergent to protein ratio in the two cases, I7-OR could be only partly unfolded in the cell extract but fully unfolded in the membrane preparation.

All together results of Figure 3 and Figure 4 suggest the presence of full-length recombinant I7-OR protein in the membranes of engineered *Hfx. volcanii*, whereas in the only previous study with the same microorganism as a host for the production of eukaryotic GPCRs, massive protein degradation of recombinant receptors was reported [16]. The ability of *Hfx. volcanii* in expressing a full-length I7-OR protein could depend on the fact that the cytoplasmic loops of the protein here produced are smaller than those of the other GPCRs previously expressed.

The present successful attempt to use *Hfx. volcanii* as a tool for producing a mammalian olfactory receptor suggests that the experimental approach adopted in the present study can be extended to other members of the GPCR family.

The right localization of the expressed OR on the plasma membrane of the transformed archaeon is very important for the function of the receptor protein. Infact ORs, which are seven helical transmembrane proteins, require a lipid environment to maintain their native conformation and should be integrated into membrane to have a right functioning, so that odorants are able to bind OR proteins by the aid of interactions, such as electrostatic bonding and van der Waals interaction, in order to trigger the olfactory signal transduction cascade.

**Figure 5:**
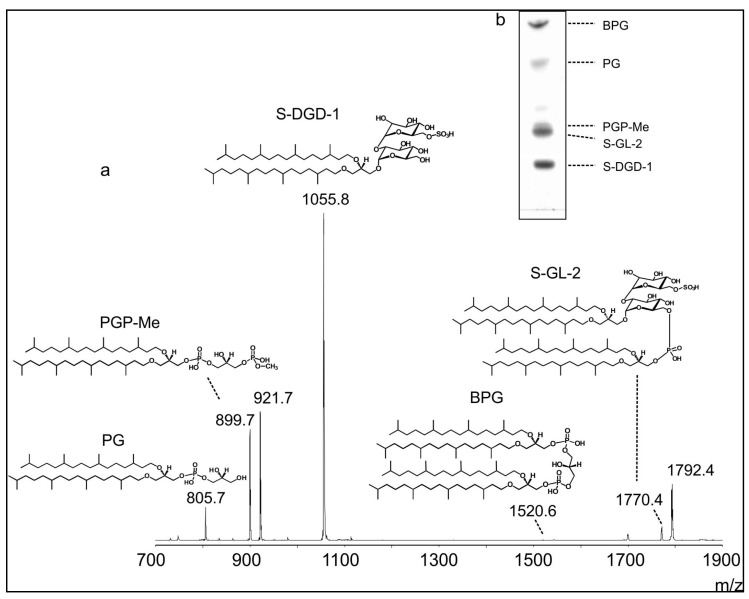
Lipid profile of *Hfx. volcanii* membranes containing ORs. (**a**) Negative MALDI-TOF mass spectrum of “intact” membranes. Lipid assignments of the main *m*/*z* signals detected in the spectrum together with the lipid structures are reported; (**b**) HPTLC chromatogram of the total lipid extract of the isolated membranes. Lipid bands on the plate are indicated by their abbreviations. BPG, bisphosphatidylglycerol (diphytanylglycerol ether analogue); PG, phosphatidylglycerol (diphytanylglycerol ether analogue); PGP-Me, phosphatidylglycerophosphate methyl ester (diphytanylglycerol ether analogue); S-DGD-1, monosulphated diglycosyl diphytanylglycerol diether; S-GL-2, glycosyl-cardiolipin.

In order to obtain detailed information about the lipid environment of the ORs produced by transformed *Hfx. volcanii* strain, the lipids of isolated membranes were analyzed by MALDI-TOF/MS and HPTLC (Figure 5).

Lipids of intact engineered membranes have been directly analyzed by MALDI-TOF/MS analysis, following a procedure recently described [32]. As mentioned before, like many extremely halophilic archaea, the membrane polar lipids of *Hfx. volcanii* are diphytanylglycerol diether lipid derivatives [24]. The major signal present in the MALDI-TOF mass spectrum at *m*/*z* 1055.8 corresponds to the monosulfated diglycosyl diphytanylglycerol diether (S-DGD-1), the most abundant glycolipid in the genus *Haloferax* [33]. The peaks at *m*/*z* 805.7, 899.7 and 921.7 are instead attributed to the phospholipid diether analogues of phosphatidylglycerol (PG), phosphatidylglycerophosphate methyl ester (PGP-Me) and its sodium adduct, respectively. The two peaks at *m*/*z* 1770.4 and 1792.4 present in the higher *m/z* range of the mass spectrum can be assigned to the glycosyl-cardiolipin S-GL-2 and its sodium adduct, respectively, previously described in *Hfx. volcanii* membranes [34]. Finally, the small peak at *m*/*z* 1520.6 correspond to the diphytanylglycerol diether analogue of the bisphosphatidylglycerol (BPG), the ubiquitous cardiolipin analog of the Archaea [35,36]. In addition, lipids were extracted from engineered membranes by standard analytical procedures and analyzed by MALDI-TOF/MS and HPTLC. The MALDI-TOF/MS analysis of the lipid extract of membranes (not shown) give rise to a lipid profile very similar to that of the intact membranes.

Figure 5b shows a typical HPTLC chromatogram of the lipid extract of *Hfx. volcanii* membranes, together with the assignments of the different individual lipid species. Individual polar lipids were identified by comparison of their retention factor values with those of archaeal lipid standards, by their responses to specific lipid staining and by MALDI-TOF mass analyses of purified lipid components (not shown). Five polar lipid bands are visible in the HPTLC plate: the most abundant lipids present in the TLC lipid profile are the glycolipid S-DGD-1 together with the two cardiolipins S-GL-2 and BPG; smaller amounts of the phospholipids PG and PGP-Me are also present in the lipid profile of the isolated membranes.

As mentioned before, the first requirement to develop bioelectronic noses is the immobilization of ORs in a manner to preserve their function. In this study, we have isolated lipid vesicles bearing ORs by disrupting the membrane of the cells where receptors have been expressed. In this way, the receptor remains in its native membrane environment, which obviates the risk of receptor alternation or activity loss, which may occur when GPCRs are expressed in heterologous cells, isolated and then reconstituted in proteoliposomes. In particular, when the archaeal cells were disrupted, the membrane fraction was isolated in the form of microsomes, *i.e.*, membrane vesicles with heterogeneous sizes. The membrane fraction in the form of archaeonanosomes, having smaller diameters, could be obtained by additional sonication of microsomes, as previously described [37].

Several biosensors based on immobilized cellular membrane fractions (nanosomes) carrying ORs have been published [37,38,39]. To our knowledge, this is the first study in which archaeal membrane domains (*i.e.*, archaeonanosomes) containing ORs have been isolated.

The future functionalization of SWNTs and graphene sheets with archaeal membranes containing ORs, here produced for the first time, could lead to a new class of hybrid materials, which combine the unique functionalities of both the components resulting in unprecedented properties.
